# Supplementary material for: The diverse liver viromes of Australian geckos and skinks are dominated by hepaciviruses and picornaviruses and reflect host taxonomy and habitat
Source: Virus Evol. 2024 May 28;10(1):veae044. doi: 10.1093/ve/veae044 (PMC11160328; doi:10.1093/ve/veae044)
Supplement: veae044_Supp [file veae044_supp.zip › suppl_data/Mahar.Supplementary Information.docx]

**SUPPLEMENTARY INFORMATION**

**Table S1.** Details of sampled lizard livers in each library.

**Table S2.** Viral data sets used in the phylogenetic analyses.

**Table S3**. Details of assembled likely vertebrate-infecting viral nucleotide consensus sequences containing an RdRp (or capsid in the case of the *Iridoviridae*).

**Figure S1**. Viral abundance across libraries. (A) Abundance (left) and relative abundance (right) of likely vertebrate-infecting viruses classified at the virus genus level in each library. Plots classified at the virus family level are presented in Figure 1. (B) Abundance of likely vertebrate-infecting viruses (green) compared to viruses likely infecting the microbiome or potential environmental contaminants (i.e., non-vertebrate viruses; yellow). Library names are as follows: Cam_M, *Carlia amax (collected from a mesic environment)*; Cmun_M, *Carlia munda* (mesic), Csex_M, *Carlia sexdentata* (mesic); Cmet_M, *Cryptoblepharus metallicus* (mesic); Gnan_A, *Gehyra nana* (arid); Gnan_M *Gehyra nana* (mesic); Garn_M, *Gehyra arnhemica* (mesic); Hbin_A, *Heteronotia binoei* (arid); Hbin_M, *Heteronotia binoei* (mesic); Hplan_A, *Heteronotia planiceps* (arid); Omar_M, *Oedura marmorata* (mesic); and are plotted taxonomically, with host order and family names in grey bars (Dip=Diplodactylidae).

**Figure S2.** Correlation between the number of individuals in each library and viral abundance and diversity. Points are coloured by host family. P-values <0.05 were considered significant and are indicated with an asterisk.

**Figure S3.** Viral abundance and alpha diversity according to host taxonomy (at the host family level). Points are coloured by host family. P-values for host taxonomy in best fit models are specified. P-values <0.05 were considered significant and are indicated with an asterisk. A p-value of “n.a” indicates that taxonomy was not in the best fit model.

**Figure S4.** Viral abundance and alpha diversity by host habitat. Points are coloured by host family. P-values <0.05 were considered significant and are indicated with an asterisk.

**Figure S5.** Beta diversity of viruses in different hosts and habitats. NMDS plots coloured by considered factors – host family and host habitat. Overlapping points are depicted by a half circle. P-values <0.05 were considered significant and are indicated with an asterisk.
