# Supplementary material for: The diverse liver viromes of Australian geckos and skinks are dominated by hepaciviruses and picornaviruses and reflect host taxonomy and habitat
Source: Virus Evol. 2024 May 28;10(1):veae044. doi: 10.1093/ve/veae044 (PMC11160328; doi:10.1093/ve/veae044)
Supplement: veae044_Supp [file veae044_supp.zip › suppl_data/Mahar.Table S2.docx]

**Table S2.** **Viral data sets used in the phylogenetic analyses.**

| **Group name** | **Figure No.** | **No. taxa** | **Alignment length before trimAl** | **Alignment length used for tree (post trimAl)** |
| --- | --- | --- | --- | --- |
| Amnoonviridae | 4 | 14 | 615 | 512 |
| Arenaviridae | 4 | 74 | 1,784 | 1,319 |
| Astroviridae | 3 | 91 | 574 | 525 |
| Bornaviridae | 4 | 25 | 963 | 947 |
| Caliciviridae | 3 | 66 | 598 | 568 |
| Flaviviridae | 2 | 231 | 913 | 502 |
| Hepacivirus only | 2 | 88 | 608 | 554 |
| Iridoviridae | 5 | 27 | 523 | 451 |
| Picornaviridae | 3 | 177 | 656 | 479 |
| Rhabdoviridae | 4 | 230 | 1,422 | 774 |
