# Supplementary figures and images for: The diverse liver viromes of Australian geckos and skinks are dominated by hepaciviruses and picornaviruses and reflect host taxonomy and habitat
Source: Virus Evol. 2024 May 28;10(1):veae044. doi: 10.1093/ve/veae044 (PMC11160328; doi:10.1093/ve/veae044)

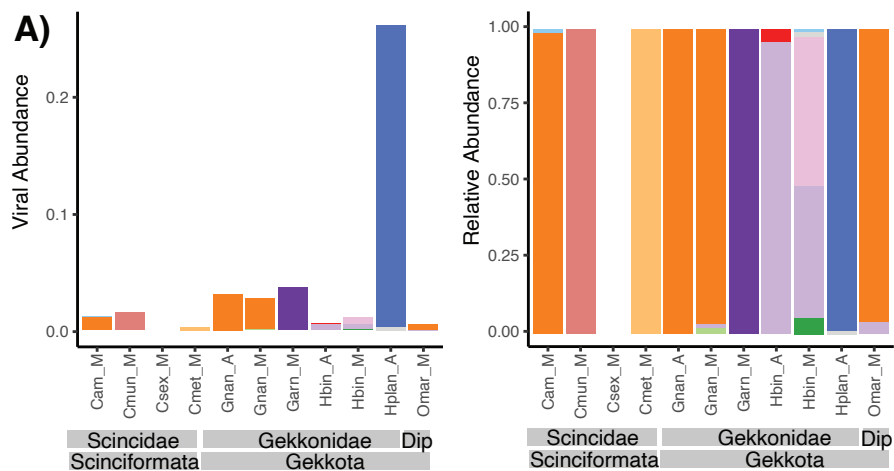

**Family - Genus**

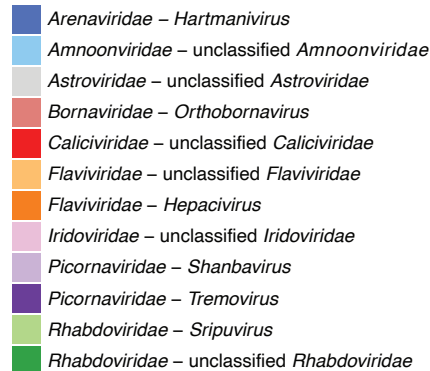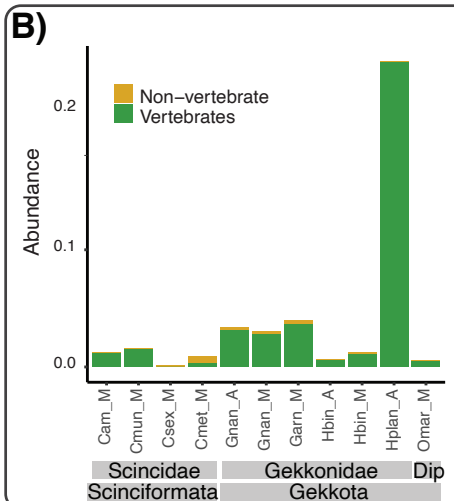

**Figure S1**

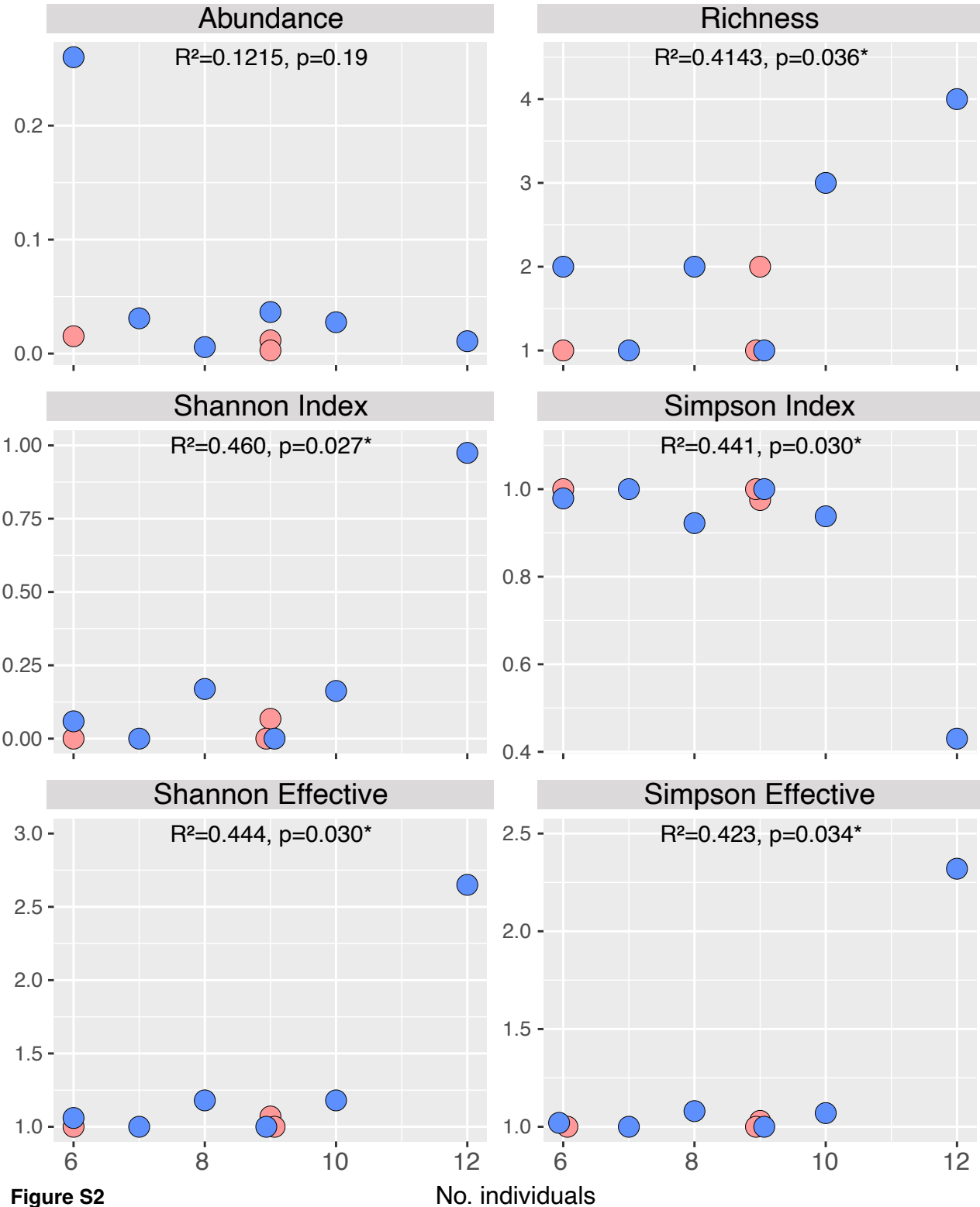

Figure S2

No. individuals

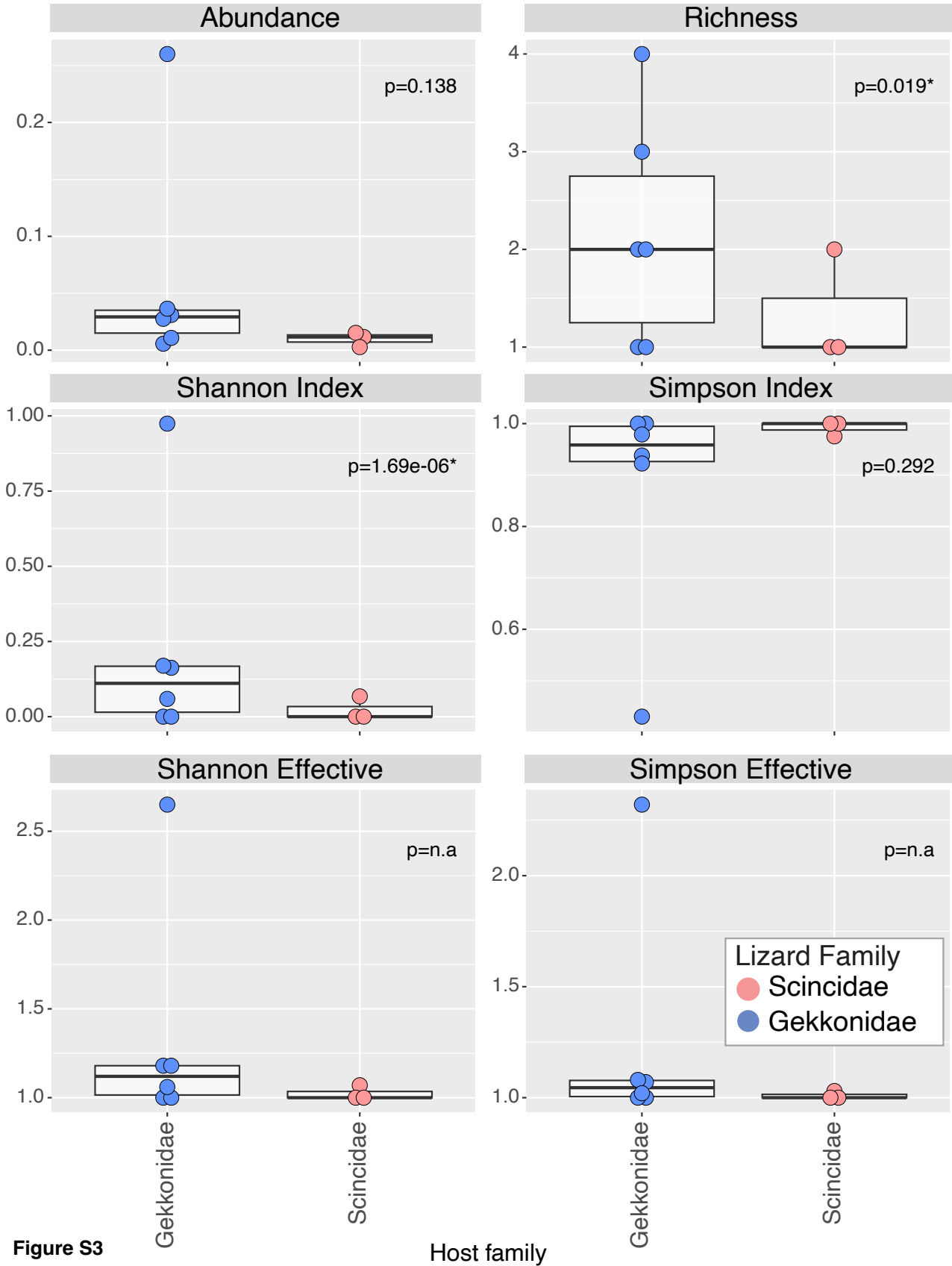

Figure S3

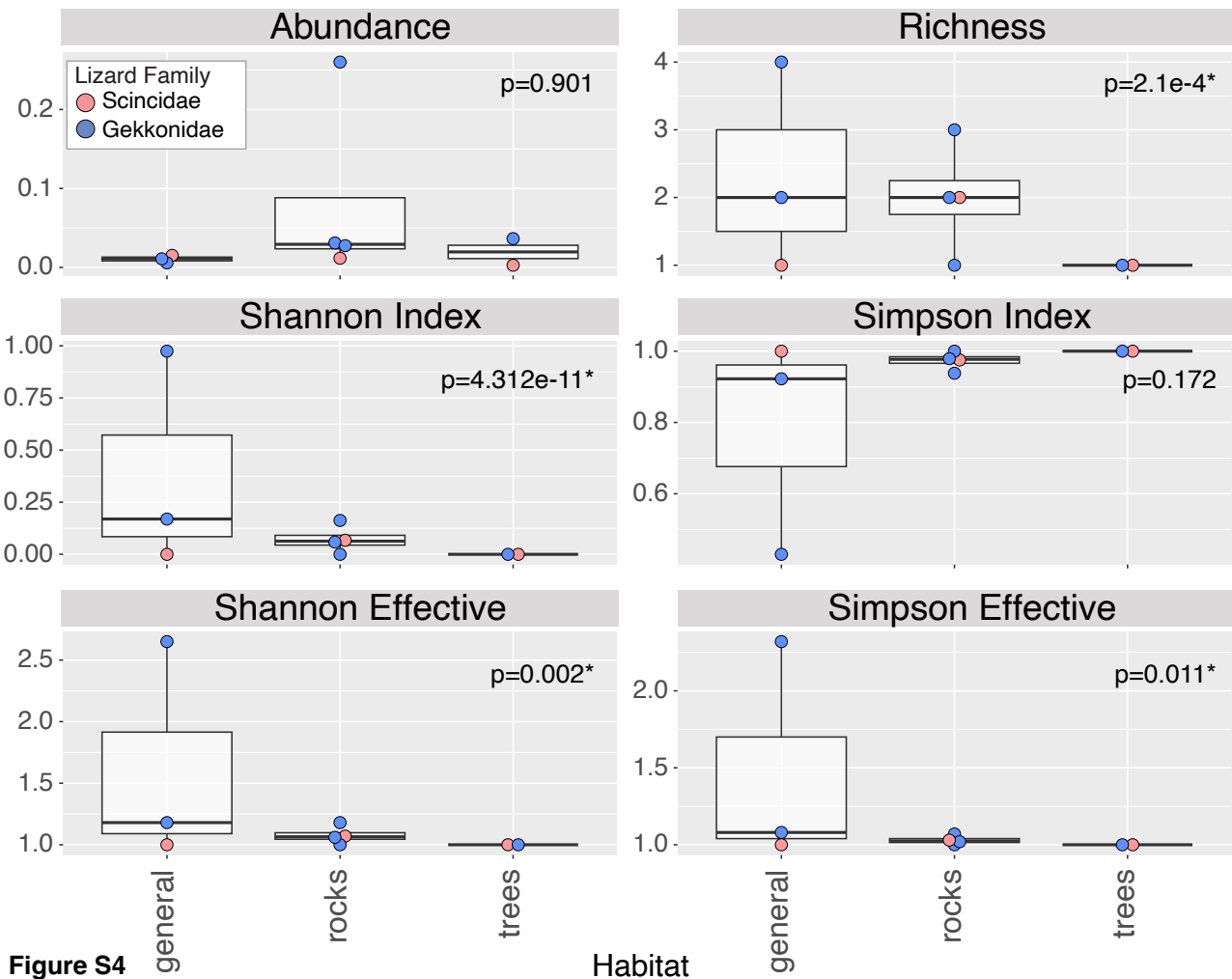

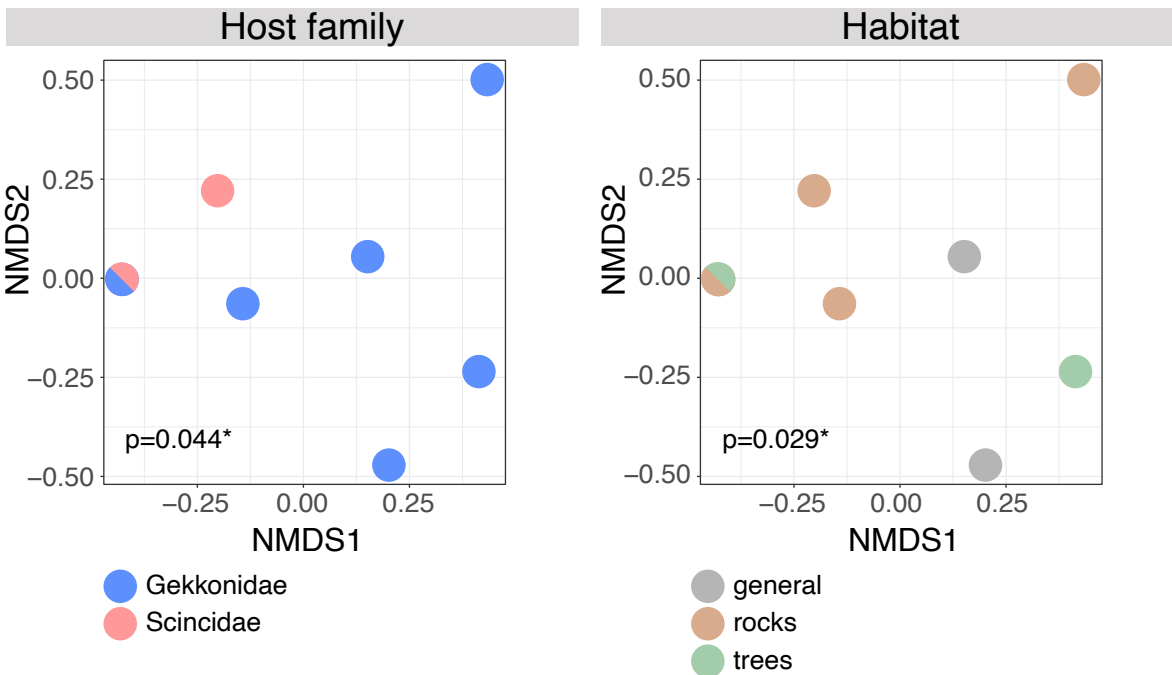

**Figure S5**

Supplement: veae044_Supp [file veae044_supp.zip › suppl_data/Mahar.Supplementary Figures.pdf]
